# Deferoxamine Intradermal Delivery Patch for Treatment of a Beta-Thalassemia Wound

**DOI:** 10.1097/AS9.0000000000000372

**Published:** 2024-01-11

**Authors:** David Perrault, Arhana Chattopadhyay, Dharshan Sivaraj, Derrick Wan, Kellen Chen, Geoffrey Gurtner, Subhro Sen

**Affiliations:** * From the Division of Plastic and Reconstructive Medicine, Stanford University School of Medicine, Stanford University, Stanford, CA; † Department of Surgery, the University of Arizona College of Medicine, Tucson, AZ.

## Abstract

Mini-abstract:

In this study, we present the first-in-human use of topical deferoxamine (DFO) in the treatment of a beta-thalassemia wound. We elected to use DFO on a patient that suffered from a chronic nonhealing wound in the setting of beta-thalassemia. Despite approximately 55 weeks of marginal improvement in healing, this patient’s wound healed completely after 21 weeks of treatment with DFO. We believe that DFO has the potential to accelerate healing in beta-thalassemia wounds through iron chelation.

## INTRODUCTION

Deferoxamine (DFO) is an iron-chelating agent that has been FDA-approved for the treatment of acute iron intoxication and chronic iron overload due to transfusion-dependent anemias for intramuscular, subcutaneous, and intravenous administration (NDA #076019). Although topical DFO has yet to be FDA-approved, it represents a promising therapy for wounds and other cutaneous problems. Topical DFO has been studied for the treatment of radiation-induced fibrosis,^1^ diabetic wounds,^2^ and sickle cell-related wounds.^3^ Its mechanism of action in the treatment of iron intoxication/overload and the related wounds is iron chelation, which reduces inflammation and oxidative stress. Additional mechanisms by which DFO may improve wound healing include HIF-1a activation and stabilization, neovascularization, and improved collagen deposition.^1,2^

DFO has been used systemically to treat iron overload diseases since 1958.^4^ Regarding wounds associated with iron overload states and chronic iron deposition, the first use of topical DFO was reported in 2018.^3^ DFO has yet to be reported for use for beta-thalassemia wounds in either humans or preclinical models. However, DFO has been used for sickle cell disease, which is similar to beta-thalassemia, in that it is a hemoglobinopathy that leads to red blood cell lysis, a subsequent need for chronic blood transfusions, and ultimately iron deposition in the skin. High levels of iron in the skin then precipitate a chronically inflamed microenvironment and skin ulceration.^5^

Given the promising preclinical data and approved clinical trial using DFO to treat sickle cell wounds, we elected to use DFO for a patient that had been suffering from a beta-thalassemia wound for 55 weeks. Her wound healed after 21 weeks of treatment.

## METHODS

The patient was a 41-year-old female with beta-thalassemia who presented with a chronic right medial ankle wound since April 2020. The wound had a long history of partially healing and recurring. On March 15, 2021, she had recurrence of the wound and presented to our care. At that time, she was treated with collagenase daily since she did not tolerate debridement. Other medical and surgical history included splenectomy, septic meningitis, endocarditis status-post bioprosthetic aortic valve replacement, mitral valve and tricuspid valve repair, and aortic root replacement. The patient had been undergoing periodic transfusions for anemia since the 1980s. She received her formal diagnosis of beta-thalassemia in 2003 and began developing leg ulcers in 2012. She underwent splenectomy in 2013. She started systemic iron chelation in 2014 and has been on multiple iron chelators since that time. Most recently she was started on deferiprone in 2018. Additionally, she has been undergoing transfusion with 2 units of packed red blood cells every 4–5 weeks.

Treatment with topical deferoxamine intradermal delivery patch was started on November 10, 2021. The treatment protocol included daily application of a new patch after cleansing the wound with unscented soap and water. The patch was secured with an atraumatic silicone dressing and kept in place at all times. The patient was instructed to keep the affected lower extremity elevated when at rest and to limit ambulation. The patient presented to the wound care center weekly for examination of the wound and debridement of any eschar down to healthy bleeding tissue. Wound size was measured with the AranzMedical Silhouette System. Briefly, the Silhouette device was held directly over the wound such that the imaging laser lines intersected at approximately the deepest and centermost portion of the wound before image capture and manual wound tracing. The wound was followed until it demonstrated completion of healing with epithelialization across the defect. At that point the treatment was stopped.

## RESULTS

**FIGURE 1.**
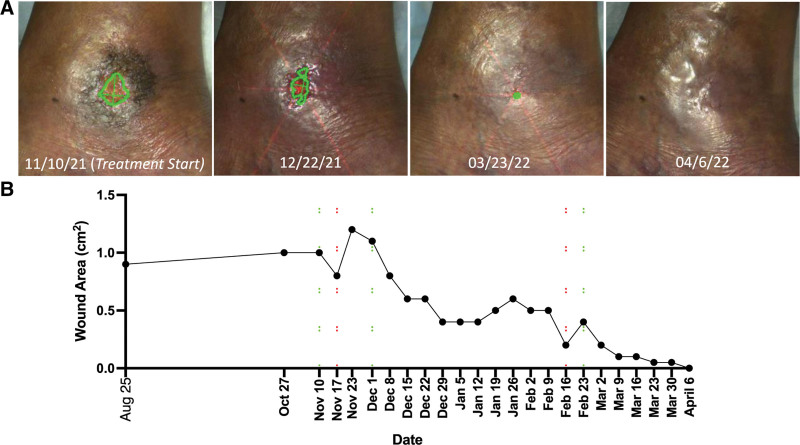
Right medial malleolus wound secondary to beta-thalassemia healed after 21 weeks of treatment with topical deferoxamine. A, Photographs with the wound outlined at select timepoints. B, Wound area plotted over time.

Before the therapy was initiated, the wound area measured 1.0 cm^2^ and wound depth measured 0.2 cm (Figs. 1A, B). This has been roughly stable in size since August 25, 2021, based on our objective photographic measurements. At this time, she also had significant discoloration of the skin surrounding the wound from chronic hemosiderin deposition. During the first week of therapy, the size of the wound decreased from 1.0 cm^2^ to 0.8 cm^2^. At that time (November 17, 2021) however, she experienced a minor rash surrounding the wound. Treatment with the deferoxamine intradermal delivery patch was paused, and she was watched closely for resolution and to ensure that the erythema was not from infection or another etiology. On November 25, 2021, the rash had resolved per the patient’s report. She restarted therapy on December 1, 2021. She then completed the remaining course of treatment without rashes, systemic symptoms, or any other adverse events. After 21 weeks of treatment, the wound had fully closed and healed (Figs. 1A, B). There was also a notable improvement in the quality of the surrounding skin, with less hemosiderin deposition based on clinical exam. The patient was seen again 9 weeks after healing, which confirmed that the wound had not recurred. As of her last visit, the patient also reports a subjective improvement in pain.

Serum was drawn throughout the course of treatment and pharmacokinetic analysis of DFO levels was performed on the samples. Systemic levels of DFO were shown to be below the quantifiable limit of 1.00 ng/mL before treatment, in the middle of treatment, and at the conclusion of treatment. Previous studies of intramuscular injection of DFO in humans at 15.24 μmol/k found that peak DFO concentration was 15.5 ± 1.8 μmol/L (or 10,184 ± 1183 ng/mL) in healthy subjects and 7.3 ± 5.3 μmol/L (or 4796 ± 3482 ng/mL) in patients with hemochromatosis.^6^ This patient had DFO levels below both that ofhealthy subjects and those with hemochromatosis recieving intramuscular injections of DFO.

## DISCUSSION

We present the first-in-human use of topical DFO in the treatment of a beta-thalassemia wound. The wound healed after 21 weeks of treatment. Additionally, the patient reported improvement in the associated pain of having an open wound. We hypothesize that the improvement in healing was a result of direct iron chelation by DFO and DFO-induced HIF-1a activation and stabilization.

The mechanism of action of DFO is extrapolated from the preclinical work done in those with sickle cell disease ulcers. As mentioned above, in the treatment of wounds, DFO has primarily been studied in the context of diabetes and sickle cell-related wounds.^3^ In diabetic wounds, DFO has been applied with success in preclinical studies and typically combined with a hydrogel wound dressing or another therapeutic compound such as polyglycolic acid/silk fibroin nanofibrous scapholds,^7^ calcium ion cross-linked sodium alginate hydrogels with copper nanoparticles,^8^ or hydrogels containing bioglass.^9^ In diabetic wounds, DFO has been shown to increase wound contraction, increase collagen density, reduce free radical formation, improve neovascularization, and increase HIF-1a.^10^ Topical DFO has yet to be used to treat diabetic wounds in humans.

Limitations of this report include that this was a single-patient case study. A randomized clinical trial to elucidate the role of topical DFO in iron-overloaded wounds should be conducted.

In conclusion, DFO has the potential to accelerate healing in beta-thalassemia wounds by iron chelation.^3^ Despite approximately 55 weeks of marginal improvement in healing before treatment, this patient’s wound healed after 21 weeks of treatment with topical DFO.
